# Propensity for Calcification in Serum Associates With 2-Year Cardiovascular Mortality in Ischemic Heart Failure With Reduced Ejection Fraction

**DOI:** 10.3389/fmed.2021.672348

**Published:** 2021-06-18

**Authors:** Marija Bojic, Lorenz Koller, Daniel Cejka, Alexander Niessner, Bernhard Bielesz

**Affiliations:** ^1^Division of Nephrology and Dialysis, Department of Medicine III, Medical University of Vienna, Vienna, Austria; ^2^Division of Cardiology, Department of Medicine II, Medical University of Vienna, Vienna, Austria; ^3^Department of Medicine III, Nephrology, Transplantation Medicine, Rheumatology, Geriatrics, Ordensklinikum Linz Elisabethinen, Linz, Austria

**Keywords:** calcification propensity, heart failure with reduced ejection fraction (HFrEF), kidney failure, chronic kidney disease, cardiovascular, mortality, vascular calcifcation, T_50_ test

## Abstract

**Background:** The propensity of serum to calcify, as assessed by the T_50_-test, associates with mortality in patients with chronic kidney disease. In chronic heart failure, phosphate and fibroblast growth factor-23 (FGF-23), which are important components of the vascular calcification pathway, have been linked to patient survival. Here, we investigated whether T_50_ associates with overall and cardiovascular survival in patients with chronic heart failure with reduced ejection fraction (HFrEF).

**Methods:** We measured T_50_, intact and c-terminal FGF-23 levels in a cohort of 306 HFrEF patients. Associations with overall and cardiovascular mortality were analyzed in survival analysis and Cox-regression models.

**Results:** After a median follow-up time of 3.2 years (25th−75th percentile: 2.0–4.9 years), 114 patients (37.3%) died due to any cause and 76 patients (24.8%) died due to cardiovascular causes. 139 patients (45.4%) had ischemic and 167 patients (54.6%) had non-ischemic HFrEF. Patients with ischemic HFrEF in the lowest T_50_-tertile had significantly greater 2-year cardiovascular mortality compared to patients in higher tertiles (*p* = 0.011). In ischemic but not in non-ischemic HFrEF, T_50_ was significantly associated with cardiovascular mortality in univariate (*p* = 0.041) and fully adjusted (*p* = 0.046) Cox regression analysis. Significant associations of intact and c-terminal FGF-23 with all-cause and cardiovascular mortality in univariate Cox regression analysis did not remain significant after adjustment for confounding factors.

**Conclusion:** T_50_ is associated with 2-year cardiovascular mortality in patients with ischemic HFrEF but not in non-ischemic HFrEF. More research on the role of T_50_ measurements in coronary artery disease is warranted.

## Introduction

Cardiovascular disease is a leading cause of death worldwide. There are many established risk factors for cardiovascular disease, of which chronic kidney disease (CKD) is known to be a main promotor of vascular aging. Cardiovascular calcification in CKD is a component of the so-called “Chronic Kidney Disease – Mineral and Bone Disorder” (CKD-MBD) syndrome, which additionally comprises abnormalities of hormones and minerals (especially fibroblast growth factor 23 (FGF-23), phosphate, calcium, parathyroid hormone, and vitamin D) and renal osteodystrophy (1). While disturbances of the CKD-MBD-axis are well-recognized to associate with higher cardiovascular disease burden in patients with kidney disease, such relationships have only recently been investigated in chronic heart failure patients. Specifically, it has been shown that levels of serum phosphate and FGF-23 associate with cardiovascular events and mortality in patients with heart failure (2–4). While it does not directly contribute to vascular calcification, FGF-23 causes cardiac hypertrophy (5). It contributes to pathological cardiac remodeling with the induction of cardiac fibrosis, ultimately leading to heart failure (6, 7).

During the calcification process in blood, calcium-phosphate crystal formation is inhibited by a number of factors such as fetuin A, pyrophosphate or magnesium by formation of protein-mineral complexes which are called “calciprotein particles” (CPPs) (8). The initial (“primary”) CPPs are small (6–50 nm), spherical and amorphous (9). In the further “ripening” process, the primary CPPs undergo topological rearrangement, become larger (several hundred nm) and transform into an ellipsoid, stable, less soluble, crystalline structure, called “secondary CPPs” (10, 11). The major role of CPPs is to transport mineral in blood to the teeth and bones for remodeling and prevent its deposition in soft tissue such as the vasculature or cardiac valves (12). CPP levels are elevated in procalcific milieus and secondary CPPs induce calcification of vascular smooth muscle cells (13–15). Furthermore, it has been shown that CPPs induce FGF-23 expression, suggesting a crucial role in cardiovascular disease (16, 17). To our knowledge, there are no studies on CPPs in the setting of heart failure, but it is known that vascular calcification along with increased arterial stiffness and consecutively reduced vessel compliance leads to left ventricular hypertrophy (due to increased afterload), fibrosis and heart failure (18). Blood calcification propensity may represent a direct link to development and maintenance of vascular damage resulting in accelerated calcification, which is a major component of cardiovascular disease.

A novel blood test (T_50_ test) measures the calcification propensity as the half-maximum transition time from primary to secondary CPPs after supersaturating patient serum with standardized amounts of calcium and phosphate and is expressed in minutes (T_50_) (19). The T_50_ test is a functional test, where a long transition time (i.e., long T_50_) indicates an intact calcification resistance, whereas an accelerated transition time (i.e., short T_50_) indicates increased calcification propensity. According to data from our group in a cohort of CKD stage I-V patients not on dialysis, T_50_ associates with phosphate, fetuin A, magnesium and also FGF-23 independently of excretory renal function after multivariate adjustment (20). T_50_ strongly associates with death and cardiovascular outcomes in CKD patients and renal transplant recipients (21–28). It has been recently shown, that T_50_ was associated with cardiovascular mortality in the large general-population based Prevention of Renal and Vascular End-Stage Disease (PREVEND-) Study (29). To our knowledge, a potential role of T_50_ in patients with chronic heart failure has not been studied so far. While the classical risk factors of chronic heart failure are known to be coronary heart disease, hypertension, valvular heart disease, obesity, smoking and diabetes (30), we aimed to look into the relevance of serum calcification propensity in this group of patients. We hypothesized that low T_50_ values are associated with survival in heart failure patients.

## Materials and Methods

### Study Population

We analyzed a cohort of chronic heart failure patients with reduced ejection fraction (HFrEF) who were recruited at the outpatient department for heart failure at the Medical University of Vienna. Upon routine outpatient clinic visits, patients who had been clinically stable for more than 4 weeks, were invited to participate in the primary, observational prospective study which was initially designed to investigate cellular markers measured by flow-cytometry (e.g., CD4^+^CD28^−^ T-cells and regulatory T cells) and serum markers including high sensitive C-reactive protein, interleukin 6, soluble urokinase plasminogen activator receptor or soluble ST2 and survival in patients with heart failure (31). Clinically stable was defined as absence of acute cardiac decompensation, hospitalization due to HFrEF, significant worsening of HFrEF, acute renal failure and acute infection. Inclusion criteria were defined as New York Heart Association functional classification (NYHA) ≥2 and the presence of either an N-terminal pro-B-type natriuretic peptide (NT-proBNP) level of ≥500 pg/mL or an echocardiographic left ventricular ejection fraction (LVEF) <40% at time of enrolment. Exclusion criteria were defined as age <18 years, presence of a severe life-threatening condition other than heart failure (e.g., malignancies), chronic inflammatory diseases, and refusal of informed consent. Diagnosis and treatment were in accordance with current heart failure guidelines (32, 33). Data on patients' characteristics, comorbidities, routine laboratory (i.e., NT-proBNP, creatinine, estimated glomerular filtration rate, serum phosphate) and echocardiography findings were collected from patients' medical charts. Ischemic heart failure was defined as a history of myocardial infarction with accompanied reduction of left ventricular ejection fraction (LVEF <40%) and typical signs and symptoms of HFrEF. In addition, patients with advanced coronary artery disease (e.g., severe 3-vessel disease) were classified as ischemic HFrEF, unless another cause was more likely to be the underlying cause for impairment of ejection fraction. Non-significant epicardial stenosis or peripheral coronary artery stenosis were not considered as ischemic HFrEF. Non-ischemic HFrEF was defined as any other underlying etiology including hypertensive HFrEF, HFrEF due to valve disease, HFrEF following myocarditis, idiopathic dilatative cardiomyopathy and others.

### Outcomes

Outcome data were collected by scanning the national death registry (Statistik Austria) and crosschecking the source data in the local electronic clinical database. Death certificates of deceased patients were used to classify into cardiovascular and non- cardiovascular cause of death. Cardiovascular mortality was defined as death due to progressive heart failure, sudden cardiac death, death due to atherosclerotic events including fatal myocardial infarction and other causes of death because of cardiovascular diseases. In the case of uncertainty, classification of cause of death (cardiovascular vs. non-cardiovascular) was discussed in the workgroup (L.K. and A.N.).

### Laboratory Measurements

NT-proBNP levels were measured as previously described (31). Blood chemistry was measured on Cobas 8,000, Roche Diagnostics, Germany, in ISO 15,189 accredited clinical laboratories of the Department of Laboratory Medicine at the Medical University of Vienna. Reference ranges for blood chemistry parameters are shown in Supplementary Table 1.

### Measurement of T_50_

Serum samples which were drawn at the baseline visit were stored at −80°C and used for T_50_-measurement. The measurements were performed by Calciscon AG, Bern, Switzerland, using a CE-certified *in-vitro* diagnostic assay as described by Pasch et al. (19). Briefly, T_50_ is defined as the nephelometrically determined half-maximum transition time from primary to secondary calciprotein particles after supersaturating patient serum with calcium and phosphate. Intra- and inter-assay coefficients of variability are 4.2 and 6.8%, respectively.

### Measurement of FGF-23

C-terminal FGF-23 (cFGF-23) was measured by ELISA detecting both, intact FGF-23 and its c-terminal fragments (Immutopics, San Clemente, USA); intact FGF-23 (iFGF-23) was measured by ELISA detecting exclusively intact FGF-23 (Kainos, Japan).

### Statistical Analysis

Continuous data are presented as mean and standard deviation if approximately normally distributed or as median and 25th to 75th percentile (IQR) otherwise. Categorical data are presented as absolute count and percentages. For survival analysis T_50_-results were divided into tertiles. To evaluate between T_50_-tertiles, we performed one-way ANOVA for normally distributed continuous data, the Kruskal-Wallis test for non-normally distributed data and the chi-squared test for nominal data. Kaplan-Meier analysis with log-rank testing was used to compare survival curves according to T_50_-tertiles.

Cox proportional hazards regression analyses were performed with adjustments for the following covariates: Model 1 was adjusted for age and sex; Model 2 was additionally adjusted for smoking, systolic blood pressure, diabetes, total cholesterol, low density lipoprotein, body mass index and history of cardiovascular events; Model 3 was additionally adjusted for high sensitivity C-reactive protein, NT-proBNP, NYHA classification and estimated glomerular filtration rate (eGFR). Before entering into the regression models systolic blood pressure, body mass index, high sensitivity C-reactive protein, NT-proBNP, c-terminal and intact FGF-23 were binary log-transformed (log with base 2) to stabilize residual distributions. Therefore, reported hazard ratios reflect the impact of doubling the respective marker on the original variable's scale. T_50_ and serum phosphate were divided by their standard deviation, therefore reported hazard ratios reflect the impact of a one standard deviation increase in T_50_ or serum phosphate, respectively. Proportional hazards (PH) assumptions were evaluated using statistical tests based on scaled Schoenfeld residuals. We found no significant relationship between residuals and time. As example, the global test in the relevant fully adjusted model for 2-year cardiovascular mortality revealed a *p*-value of 0.243 indicating no violation of PH assumptions. As such, no adaption such as adding covariate/time interaction or stratification was necessary. Competing risk analysis was performed using STATA's stcrreg command based on Fine and Gray's proportional subhazards model. A two-tailed *p*-value of <0.05 was considered statistically significant. The statistical analyses were performed with IBM SPSS Statistics (Version 24) and STATA (Version 13 and 16).

## Results

### Patients' Characteristics and Outcomes

321 chronic heart failure patients with reduced ejection fraction had been recruited between January 2008 and July 2013. Stored frozen serum samples for measurement of T_50_ and FGF-23 with complete follow-up were available in 306 patients, who represent our final study population. The basic characteristics are shown in Table 1 according to T_50_-tertiles. Overall, there was no significant difference in the severity and the underlying form of HFrEF (ischemic vs. non-ischemic HFrEF) nor in other comorbidities between T_50_-tertiles. There was a significant difference in serum phosphate, albumin and iFGF-23 between T_50_-tertiles. Median T_50_ value was 291 (IQR: 218 – 356) min. One hundred thirty-nine patients (45.4%) were diagnosed with HFrEF of ischemic etiology and 167 patients (54.6%) had HFrEF of non-ischemic etiology. NT-proBNP level at baseline was 1,151 (IQR: 1777-2425) pg/ml. The kidney function was slightly reduced (mean eGFR of 80 (±14) ml/min/1,73 m^2^). The median iFGF-23 level was 64.4 (IQR: 48.0–90.2) pg/ml and the median cFGF-23 level was 39.6 (IQR: 19.4–84.1) RU/ml. The basic characteristics according to the etiology of HFrEF are shown in Supplementary Tables 2A,B.

**Table 1:** Basic characteristics by T_50_ tertiles.

	**1st Tertile**	**2nd Tertile**	**3rd Tertile**	***p*-Value**
	***N* = 102**	***N* = 102**	***N* = 102**	
T_50_ (min)	171.00 (131.25–220.50)	290.50 (271.00–318.00)	375.00 (356.00–405.50)	
Age (years)	65 (55–71)	63 (56–71)	66 (58–72)	*P* = 0.149
Males	90 (88.2)	79 (77.5)	80 (78.4)	*P* = 0.091
**Underlying form of HFrEF**				
Ischaemic HFrEF	41 (40.2)	47 (46.1)	51 (50)	*P* = 0.367
Non-ischaemic HFrEF	61 (59.8)	55 (53.9)	51 (50)	
**NYHA-class**				
II	54 (52.9)	62 (60.8)	52 (51)	*P* = 0.555
III	45 (44.1)	38 (37.3)	48 (47.1)	
IV	3 (2.9)	2 (2.0)	2 (2.0)	
**LVEF**				
Mild	30 (29.4)	32 (31.4)	39 (38.2)	*P* = 0.457
Moderate	33 (32.4)	39 (38.2)	33 (32.4)	
Severe	39 (38.2)	31 (30.4)	30 (29.4)	
**Comorbidities**				
Hypertension	79 (77.5)	76 (74.5)	81 (79.4)	*P* = 0.703
Atrial fibrillation/flutter	44 (43.1)	42 (41.2)	45 (44.1)	*P* = 0.911
Previous MCI	35 (34.3)	46 (45.1)	46 (45.1)	*P* = 0.196
PAD	16 (15.7)	15 (14.7)	20 (19.6)	*P* = 0.610
Previous stroke or TIA	8 (7.8)	12 (11.8)	7 (6.9)	*P* = 0.426
Hyperlipidaemia	64 (62.7)	69 (67.6)	70 (68.6)	*P* = 0.635
Diabetes mellitus	47 (46.1)	36 (35.3)	32 (31.4)	*P* = 0.08
Previous or active smoker	79 (77.5)	76 (74.5)	74 (72.5)	*P* = 0.719
**Laboratory parameters**				
Creatinine (mg/dl)	1.27 (0.99–1.73)	1.14 (0.95–1.43)	1.17 (1.00–1.41)	*P* = 0.107
eGFR, MDRD (ml/min/1.73 m^2^)	58.61 (41.92–79.05)	63.49 (49.03–79.90)	65.81 (48.12–77.30)	*P* = 0.320
eGFR, CKD-EPI (ml/min/1.73 m^2^)	77.23 (65.82–88.57)	81.45 (70.98–90.53)	78.91 (70.53–88.33)	*P* = 0.441
NT-proBNP (pg/ml)	1,383.00 (633.38–3,012.50)	1221.00 (484.28–2643.25)	902.30 (388.20–2014.75)	*P* = 0.100
Phosphate (mmol/l)	1.19 (1.02–1.35)	1.06 (0.95–1.17)	0.97 (0.84–1.09)	***P*** **<** **0.001**
Total calcium (mmol/l)	2.43 (2.35–2.53)	2.43 (2.37–2.48)	2.45 (2.39–2.51)	*P* = 0.242
Albumin (g/l)	42.80 (39.73–45.13)	43.90 (41.70–45.40)	44.30 (41.60–45.90)	***P*** **=** **0.024**
iFGF−23 (pg/ml)	69.05 (51.50–109.98)	63.50 (46.73–83.95)	62.50 (43.53–82.68)	***P*** **=** **0.021**
cFGF−23 (RU/ml)	51.55 (22.55–154.03)	35.55 (19.00–80.43)	34.05 (17.85–77.15)	*P* = 0.05
Magnesium (mmol/l)	0.82 (0.75–0.90)	0.83 (0.76–0.87)	0.84 (0.80–0.89)	*P* = 0.137
CRP (mg/dl)	0.40 (0.20–0.81)	0.34 (0.14–0.56)	0.26 (0.12–0.65)	*P* = 0.069

*Values are presented as median (interquartile range) or n (%). Reference ranges for blood chemistry parameters are shown in Supplementary Table 1. cFGF-23, C-terminal fibroblast growth factor 23; iFGF-23, intact fibroblast growth factor 23; HFrEF, heart failure with reduced ejection fraction; CRP, C-reactive protein; eGFR, CKD-EPI: estimated glomerular filtration rate using the Chronic Kidney Disease Epidemiology Collaboration-formula; eGFR, MDRD: estimated glomerular filtration rate using the Modification of Diet in Renal Disease-formula; MCI: myocardial infarction; NT-proBNP, N-terminal pro B-type natriuretic peptide; NYHA, New York Heart Association functional classification; LVEF, reduced left ventricular ejection fraction (mild:40–50% ejection fraction; moderate: 30–40% ejection fraction; severe: <30% ejection fraction); PAD, peripheral artery disease; T_50_, serum calcification propensity expressed by the half-maximum transition time from primary to secondary calciprotein particles as measured by nephelometry; TIA, transient ischemic attack. Bold values are statistically significant*.

During a median follow-up of 3.2 (IQR: 2.0–4.9) years, 114 patients (37.3%) reached the primary endpoint of death due to any cause of which 76 patients (24.8%) died from a cardiovascular event. No patients were lost to follow up.

### T_50_ and Mortality

**Figure 1:**
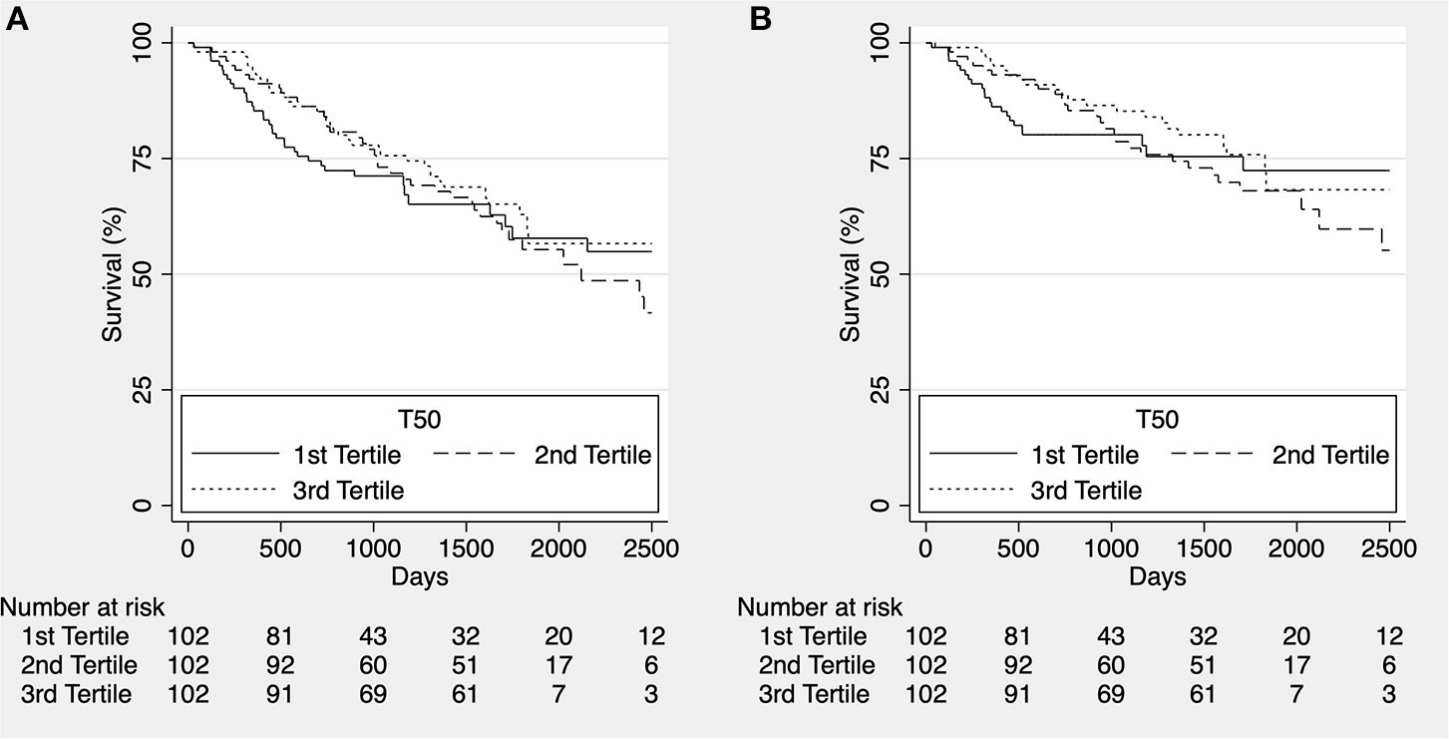
Kaplan-Meier estimates for overall survival [**(A)**, *p* = 0.636] and cardiovascular survival [**(B)**, *p* = 0.582] according to T_50_ tertiles for the whole cohort in complete follow up.

In the complete follow up there was no difference in overall or cardiovascular survival between T_50_ tertiles (*p* = 0.636 and *p* = 0.582, respectively; Figures 1A,B).

**Figure 2:**
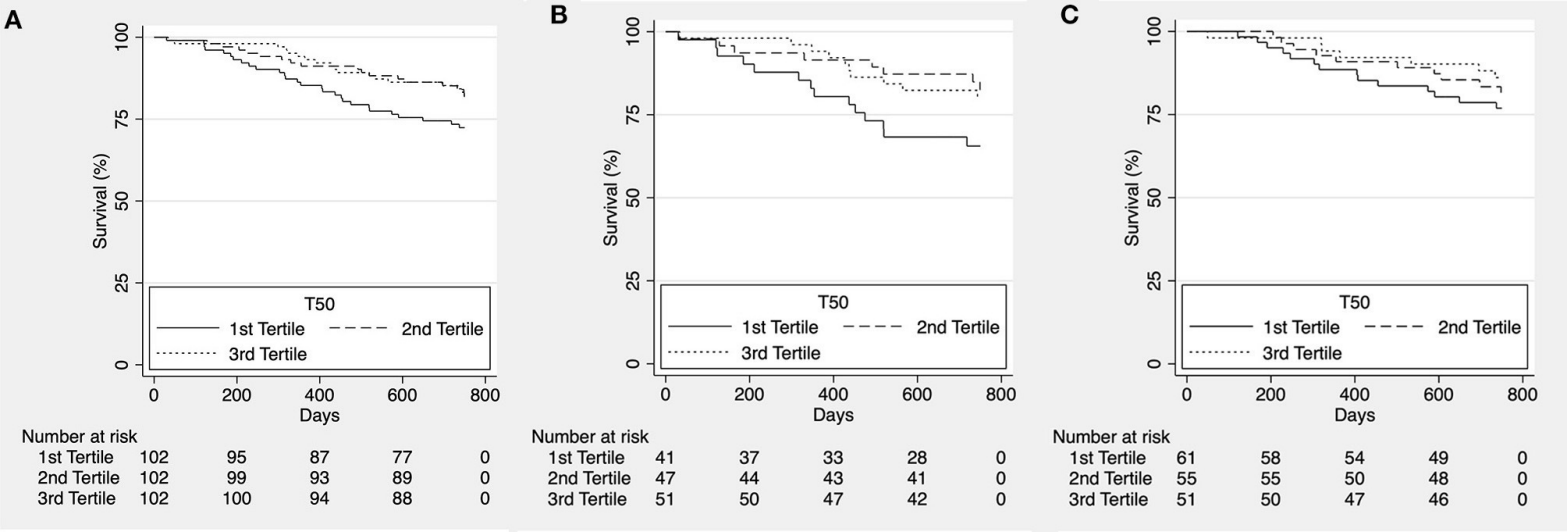
Kaplan-Meier estimates for overall survival according to T_50_ tertiles for the whole cohort [**(A)**, *p* = 0.068], patients with ischemic heart failure with reduced ejection fraction [HFrEF; **(B)**, *p* = 0.095] and patients with non-ischemic HFrEF [**(C)**, *p* = 0.434].

**Figure 3:**
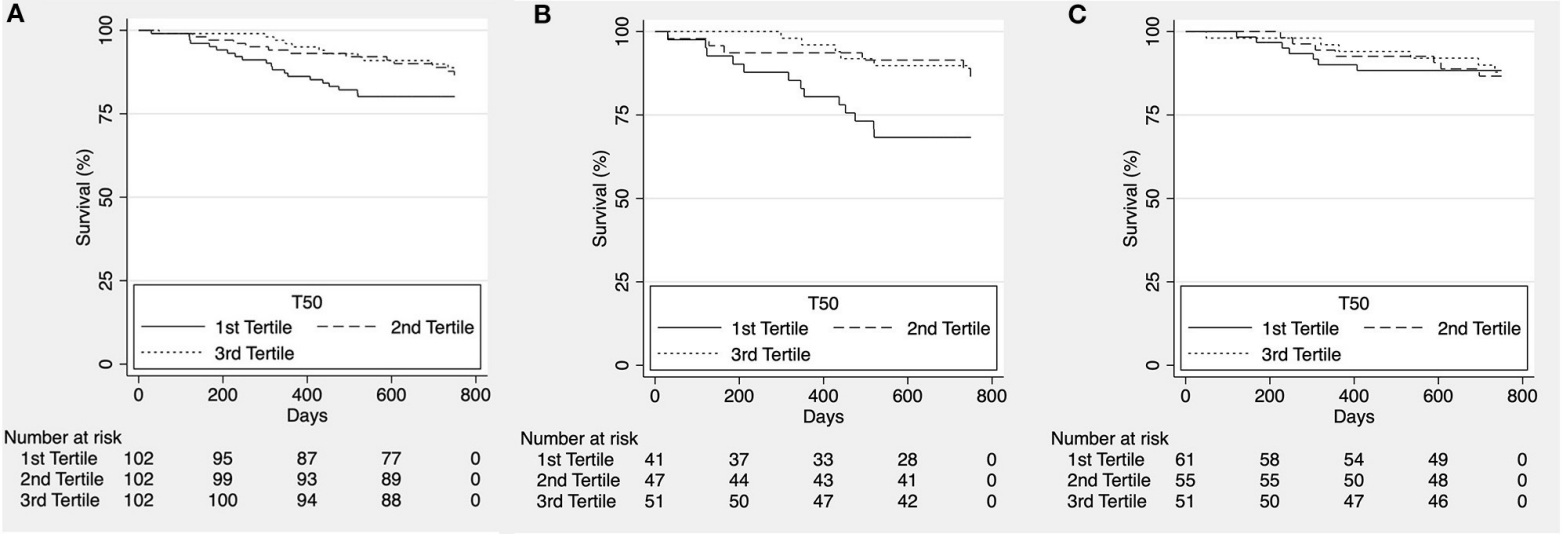
Kaplan-Meier estimates for cardiovascular survival according to T_50_ tertiles for the whole cohort [**(A)**, *p* = 0.101], patients with ischemic heart failure with reduced ejection fraction [HFrEF; **(B)**, *p* = 0.011] and patients with non-ischemic HFrEF [**(C)**, *p* = 0.978].

To assess potential shorter-term effects of increased calcification propensity, we reanalyzed the data after censoring at ~2 years (750 days). While we did observe a significant trend for higher all-cause (Figure 2A) and cardiovascular mortality (Figure 3A) in patients in the lower T_50_-tertiles (i.e., increased calcification propensity) (p for trend = 0.036 and 0.04, respectively), there was no significant difference in overall all-cause or cardiovascular mortality between T_50_ tertiles in log-rank analysis (*p* = 0.068 and *p* = 0.101, respectively).

We then subdivided the patients according to etiology of heart failure into a group of ischemic and non-ischemic causes for HFrEF. No difference was observed for all-cause mortality (*p* = 0.095; Figure 2B and *p* = 0.434; Figure 2C, respectively) however, cardiovascular mortality was significantly higher in lower T_50_ tertiles in the group of HFrEF patients of ischemic etiology (*p* = 0.011; Figure 3B) but not in the group of HFrEF patients of non-ischemic etiology (*p* = 0.978; Figure 3C).

To further characterize our findings, we performed Cox regression analyses (Table 2). In line with the survival data, there was no association between T_50_ and all-cause or cardiovascular mortality in the whole cohort. However, lower T_50_-values were associated with cardiovascular mortality in the subgroup of patients with ischemic HFrEF both in univariate analysis (*p* = 0.041) and in the adjusted model (*p* = 0.046). No associations were observed in the non-ischemic group of patients. The latter findings were verified by competing risk analysis of cardiovascular mortality competing with death due to any other cause using the fully adjusted model in the ischemic HFrEF subgroup [subhazard ratio 0.65 (95% CI: 0.45–0.93; *p* = 0.019)].

**Table 2:** Univariate and multivariate Cox regression analysis for T_50_ and all-cause and cardiovascular mortality.

	**All-Cause Mortality**				**Cardiovascular Mortality**			
	**Univariate analysis**		**Multivariate anlaysis**		**Univariate analysis**		**Multivariate anlaysis**	
	**HR (95% CI)**	**P**	**HR (95% CI)**	**P**	**HR (95% CI)**	**P**	**HR (95% CI)**	**P**
**Overall Cohort**	0.83 (0.65–1.06)	0.138			0.83 (0.62–1.10)	0.198		
Model 1			0.82 (0.64–1.05)	0.114			0.82 (0.61–1.09)	0.174
Model 2			0.89 (0.69–1.14)	0.341			0.91 (0.68–1.23)	0.543
Model 3			0.90 (0.71–1.14)	0.383			0.93 (0.69–1.24)	0.601
**Ischemic HFrEF**	0.80 (0.55–1.15)	0.229			0.65 (0.43–0.98)	**0.041**		
Model 1			0.80 (0.56–1.14)	0.224			0.66 (0.44–0.99)	**0.047**
Model 2			0.86 (0.60–1.25)	0.429			0.70 (0.45–1.07)	0.096
Model 3			0.77 (0.54–1.10)	0.151			0.66 (0.44–0.99)	**0.046**
**Non-ischemic HFrEF**	0.84 (0.61–1.16)	0.292			1.00 (0.66–1.50)	0.989		
Model 1			0.85 (0.61–1.18)	0.325			1.01 (0.67–1.52)	0.967
Model 2			0.94 (0.67–1.34)	0.75			1.20 (0.78–1.83)	0.411
Model 3			1.01 (0.71–1.44)	0.954			1.39 (0.89–2.16)	0.148

*Hazard ratios reflect the impact of a one standard deviation increase in T_50_*.

*The multivariate model was adjusted for the following covariates*.

*Model 1: age and sex*.

*Model 2: age, sex, smoking, systolic blood pressure, diabetes, total cholesterol, low density lipoprotein, body mass index and history of cardiovascular events*.

*Model 3: age, sex, smoking, systolic blood pressure, diabetes, total cholesterol, low density lipoprotein, body mass index, history of cardiovascular events, high sensitivity C-reactive protein, NT-proBNP, NYHA classification and eGFR, CKD-EPI*.

*HFrEF, heart failure with reduced ejection fraction; HR, hazard ratio; T_50_, serum calcification propensity expressed by the half-maximum transition time from primary to secondary calciprotein particles as measured by nephelometry; NT-proBNP, N-terminal pro B-type natriuretic peptide; NYHA, New York Heart Association functional classification; eGFR, CKD-EPI, estimated glomerular filtration rate using the Chronic Kidney Disease Epidemiology Collaboration-formula. Bold values are statistically significant*.

### FGF-23 and Mortality

We also measured FGF-23 using two different assays (intact and c-terminal) to assess how associations of this parameter with specific endpoints relate to those of T_50_ as both parameters are specifically involved in mineral metabolism. Both, for all-cause and cardiovascular mortality, lowest survival was observed in the highest FGF-23 tertile, although this was not consistently significant in the HFrEF subgroup of ischemic etiology (Supplementary Figures 1–4).

Likewise, univariate regression analysis demonstrated significant associations of FGF-23 with all-cause and cardiovascular mortality. However, none of these effects remained significant in multivariate adjusted models independent of the assay used (Supplementary Table 3). Notably, serum phosphate was also not associated with all-cause and cardiovascular survival (Supplementary Table 3).

## Discussion

The aim of this study was to investigate the association of serum calcification propensity, as measured by the novel T_50_-test with mortality in patients with chronic heart failure with reduced ejection fraction. Our main finding is that low T_50_ values associate with 2-year cardiovascular survival in patients with ischemic HFrEF but not in those with non-ischemic HFrEF.

The T_50_ test was first developed in patients with chronic kidney disease – a state in which pronounced premature cardiovascular calcification occurs, which leads to a particularly high rate of cardiovascular morbidity and mortality in this patient population (21–25, 27, 28).

Our cohort consisted of patients with chronic heart failure with reduced ejection fraction and largely preserved renal function. The etiology of heart failure was ischemic in approximately half of the patients and rather heterogeneous in the remainder with different underlying causes, which we characterized “non-ischemic.” Naturally, pathophysiological processes will differ between these patient groups: in patients with ischemic cardiomyopathy, the coronary arteries are affected by atherosclerosis and pathological calcification – a condition that was ruled out in the patients in the “non-ischemic group.” Secondary CPPs can induce proinflammatory responses, oxidative stress and calcification of vascular smooth muscle cells (15, 34). Therefore, secondary CPPs may contribute to calcification of the coronary arterial wall. From a mechanistic point of view, it seems plausible that associations of T_50_ – which assesses the calcification propensity in serum – with cardiovascular survival appear to predominantly pertain to patients with ischemic heart failure. Consistent with this notion, it was shown in the prospective Chronic Renal Insufficiency Cohort (CRIC)-study that lower T_50_ values had been associated with more severe coronary artery calcification and its progression in patients with CKD stages 2 to 4 (28). In the Evaluation of Cinacalcet Therapy to Lower Cardiovascular Events (EVOLVE)-study, lower T_50_ values were associated with myocardial infarction and peripheral vascular events in patients receiving hemodialysis (25).

To our knowledge, our study is the first to describe such associations also in HFrEF patients with only minor renal function impairment. One could speculate that in ischemic HFrEF with relatively preserved kidney function, higher calcification propensity might be a late finding in the disease course, predominantly in patients with poorer short-term outcomes. Our patients have been treated in a tertiary cardiology center for chronic heart failure at which treatment for cardiovascular risk factors is a crucial part of standard of care. Therefore, ideally, the patients would have had an improved cardiovascular risk profile over the course of their follow-up which may have had an impact on cardiovascular and overall survival, explaining why associations of T_50_ with mortality appear to fade in the longer term. To dissect this further, consecutive measurements of T_50_ would be an interesting asset. Furthermore, T_50_ readings may lay the foundation for therapeutic interventions aiming at decreasing the propensity of serum for calcification and ultimately decreasing cardiovascular risk, although this needs to be tested in prospective trials. In a population with only slightly reduced kidney function, T_50_ might rather reflect momentary predispositions to calcification, which can be modified by treatment interventions. For example, it has been demonstrated that interventions such as oral supplementation with magnesium, lowering of serum phosphate by oral phosphate binder therapy and other interventions improve T_50_ in CKD patients, suggesting lowered propensity for calcification (35–40).

A role of mineral metabolism in heart failure has been previously suggested in studies on FGF23, which is a bone-derived hormone that plays a well-established role in CKD patients. Elevated FGF-23 levels have been associated with cardiovascular events and mortality in patients with CKD (41, 42). FGF-23 has been shown to directly induce left ventricular hypertrophy (5). Furthermore, FGF-23 levels, even if within the normal range, show a correlation with left ventricular mass, hypertrophy and geometry in a community-based cohort (43). In patients with heart failure, FGF-23 levels are elevated and predict cardiovascular events (44–46). It was noted that heart failure patients, who had significantly reduced kidney function, also had even worse outcomes (47). In our cohort of patients with relatively preserved kidney function and heart failure with reduced ejection fraction, both cFGF-23 and iFGF-23 associated with survival in univariate regression analysis but not any more in the fully adjusted model. This is in keeping with a previous finding that higher FGF-23 levels are associated with outcome in patients with heart failure with preserved ejection fraction, but not in those with HFrEF (48).

In the context of CKD-MBD, an association between hyperphosphatemia and increased mortality was first found in chronic hemodialysis patients (49). Additionally, levels of serum phosphate even if within the normal range were associated with greater left ventricular mass and with increased risk of heart failure in populations without CKD (50, 51). Serum phosphate is a major determinant of calcification propensity (19). While phosphate did differ significantly according to T_50_ tertiles, we did not observe independent associations of phosphate and mortality in our cohort. This is consistent with another cohort of prevalent chronic heart failure patients, that did not find a significant association of phosphate levels with mortality either (46). While a residual influence of kidney function on phosphate levels cannot be excluded, we did neither see a significant association of eGFR with T_50_ in the fully adjusted model. This is in line with previous data that calcification propensity is independent of excretory renal function (20). Therefore, our data suggest that measuring the overall propensity of serum for calcification (in a functional manner as with the T_50_ test) might yield improved information of clinical relevance as compared to analyzing individual components (e.g., phosphate or magnesium) of this complex system.

Our study has limitations: It is retrospective in nature as we used a historical cohort with only one T_50_ baseline measurement at inclusion. Therefore, heterogeneous effects of treatment over time on T_50_ cannot be assessed. The study cohort consisted of a mid-European Caucasian population, therefore confirmation in different ethnical groups is warranted. However, follow-up time is long with a considerable event rate of hard endpoints. Furthermore, this is – to our best knowledge – the first study investigating calcification propensity in the setting of HFrEF and our results lay the groundwork for further investigation of T_50_ in coronary artery disease.

## Conclusion

In our study cohort of patients with chronic HFrEF, T_50_ associated with 2-year cardiovascular mortality in patients with ischemic HFrEF, but not in patients with non-ischemic HFrEF suggesting a relationship of T_50_ with vascular calcification in the context of coronary artery disease. Further studies are warranted to confirm and extend our findings in the field of ischemic heart disease.

## Data Availability Statement

The raw data supporting the conclusions of this article will be made available by the authors, without undue reservation.

## Ethics Statement

The studies involving human participants were reviewed and approved by The Ethics Committee of the Medical University of Vienna (EK 2230/2017), assuring adherence to the Declaration of Helsinki. The patients/participants provided their written informed consent to participate in this study.

## Author Contributions

MB and BB: study design, acquisition of funding, and preparation of the manuscript. MB, LK, and BB: data acquisition and assessment. MB, LK, and BB: statistical analysis. MB, LK, DC, AN, and BB: data interpretation. MB, LK, DC, AN, and BB: contribution to the discussion. All authors contributed to the article and approved the submitted version.

## Conflict of Interest

The authors declare that the research was conducted in the absence of any commercial or financial relationships that could be construed as a potential conflict of interest.

## Abbreviations

cFGF-23: C-terminal FGF-23
CKD: chronic kidney disease
CKD-MBD: chronic kidney disease - mineral and bone disorder
CPPs: calciprotein particles
CRP: C-reactive protein
eGFR: estimated glomerular filtration rate
eGFR, CKD-EPI: estimated glomerular filtration rate using the Chronic Kidney Disease Epidemiology Collaboration-formula
eGFR, MDRD: estimated glomerular filtration rate using the Modification of Diet in Renal Disease-formula
FGF-23: fibroblast growth factor 23
HFrEF: heart failure with reduced ejection fraction
iFGF-23: intact FGF-23
LVEF: left ventricular ejection fraction
MCI: myocardial infarction
NT-proBNP: N-terminal pro-B-type natriuretic peptide
NYHA: New York Heart Association functional classification
PAD: peripheral artery disease
T_50_: serum calcification propensity expressed by the half-maximum transition time from primary to secondary calciprotein particles as measured by nephelometry
TIA: transient ischemic attack.
