# Supplementary material for: Propensity for Calcification in Serum Associates With 2-Year Cardiovascular Mortality in Ischemic Heart Failure With Reduced Ejection Fraction
Source: Front Med (Lausanne). 2021 Jun 18;8:672348. doi: 10.3389/fmed.2021.672348 (PMC8249741; doi:10.3389/fmed.2021.672348)
Supplement: Supplementary file 1 [file Data_Sheet_1.pdf]

Supplemental table 1: Reference ranges for blood chemistry

Laboratory parameters	Reference range (unit)
Creatinine, men	0.7-1.2 (mg/dl)
Creatinine, women	0.5-0.9 (mg/dl)
NT-proBNP	0-125 (pg/ml)
Serum phosphate	0.8-1.45 (mmol/l)
Total calcium, age 18 – 59 years	2.15-2.50 (mmol/l)
Total calcium, age 60 – 90 years	2.20-2.55 (mmol/l)
Total calcium, age > 90 years	2.05-2.40 (mmol/l)
Albumin	35-52 (g/l)
Magnesium	0.66-1.07 (mmol/l)
CRP	< 0.5 (mg/dl)

CRP: C-reactive protein; NT-proBNP: N-terminal pro B-type natriuretic peptide

Supplemental Table 2a: Basic characteristics by T_{50} tertiles in patients with ischemic HFrEF

Values are presented as median (interquartile range) or n (%). Reference ranges for blood chemistry parameters are shown in supplemental table 1.

	**1st Tertile** n=41	**2nd Tertile** n=47	**3rd Tertile** n=51	**p Value**
T_{50} (min)	171 (142-228)	291 (272-318)	379 (353-407)	
Age (years)	69 (61-73)	65 (58-72)	68 (61-77)	p=0.179
Males	38 (92.7%)	39 (83.0%)	42 (92.4%)	p=0.306
NYHA-class				
II	22 (53.7%)	26 (55.3%)	23 (45.1%)	p=0.611
III	18 (43.9%)	20 (42.6%)	28 (54.9%)	
IV	1 (2.4%)	1 (2.1%)	0 (0%)	
LVEF				
Mild	12 (29.3%)	14 (29.8%)	13 (25.5%)	p=0.27
Moderate	14 (34.1%)	18 (38.3%)	22 (43.1%)	
Severe	15 (36.6%)	15 (31.9%)	16 (31.4%)	
Comorbidities				
Hypertension	38 (92.7%)	38 (80.9%)	44 (86.3%)	p=0.273
Atrial fibrillation/flutter	18 (43.9%)	14 (29.8%)	24 (47.1%)	p=0.187
Previous MCI	32 (78.0%)	43 (91.5%)	46 (90.2%)	p=0.121
PAD	13 (31.7%)	10 (21.3%)	16 (31.4%)	p=0.445
Previous stroke or TIA	4 (9.8%)	7 (14.9%)	2 (3.9%)	p=0.175
Hyperlipidaemia	38 (92.7%)	44 (93.6%)	43 (84.3%)	p=0.244
Diabetes mellitus	28 (68.3%)	18 (38.3%)	19 (37.3%)	**p=0.004**
Previous or active smoker	30 (73.2%)	36 (76.6%)	38 (74.5%)	p=0.932
Laboratory parameters				
Creatinine (mg/dl)	1.46 (1.06-1.89)	1.25 (0.99-1.46)	1.20 (1.06-1.50)	**p=0.044**
eGFR, MDRD (ml/min/1.73 m^2)	48.92 (37.96-68.38)	61.59 (46.80-79.86)	60.75 (46.86-70.30)	p=0.065
eGFR, CKD-EPI (ml/min/1.73 m^2)	71.78 (63.56-80.10)	78.02 (69.38-89.10)	76.04 (67.10-83.21)	p=0.070
NT-proBNP (pg/ml)	1544.00 (687.60-2927.00)	1313.00 (504.50-2073.00)	1323.00 (456.70-2596.00)	p=0.687
Phosphate (mmol/l)	1.12 (1.00-1.39)	1.06 (0.97-1.16)	1.01 (0.86-1.09)	**p=0.001**
Total calcium (mmol/l)	2.42 (2.35-2.47)	2.43 (2.36-2.48)	2.45 (2.37-2.50)	p=0.411
Albumin (g/l)	41.65 (39.05-44.25)	43.85 (41.00-45.75)	43.90 (41.43-45.68)	**p=0.035**
iFGF-23 (pg/ml)	70.50 (52.95-136.50)	63.80 (51.90-82.60)	62.00 (44.70-90.90)	p=0.127
cFGF-23 (RU/ml)	57.20 (28.00-157.60)	30.10 (19.40-74.70)	35.40 (18.74-76.30)	p=0.092
Magnesium (mmol/l)	0.81 (0.74-0.86)	0.83 (0.73-0.88)	0.84 (0.79-0.90)	p=0.098
CRP (mg/dl)	0.29 (0.17-0.57)	0.28 (0.10-0.52)	0.23 (0.15-0.55)	p=0.726

cFGF-23: C-terminal fibroblast growth factor 23; iFGF-23: intact fibroblast growth factor 23; HFrEF: heart failure with reduced ejection fraction; CRP: C-reactive protein; eGFR, CKD-EPI: estimated glomerular filtration rate using the Chronic Kidney Disease Epidemiology Collaboration-formula; eGFR, MDRD: estimated glomerular filtration rate using the Modification of Diet in Renal Disease-formula; MCI: myocardial infarction; NT-proBNP: N-terminal pro B-type natriuretic peptide; NYHA: New York Heart Association functional classification; LVEF: reduced left ventricular ejection fraction (mild:40-50% ejection fraction; moderate: 30-40% ejection fraction; severe: <30% ejection fraction); PAD: peripheral artery disease; T_{50}: serum calcification propensity expressed by the half-maximum transition time from primary to secondary calciprotein particles as measured by nephelometry; TIA: transient ischemic attack

Supplemental Table 2b: Basic characteristics by T_{50} tertiles in patients with non-ischemic HFrEF

Values are presented as median (interquartile range) or n (%). Reference ranges for blood chemistry parameters are shown in supplemental table 1.

	**1st Tertile** n=61	**2nd Tertile** n=55	**3rd Tertile** n=51	**p Value**
T_{50} (min)	171 (120-215)	289 (270-318)	366 (357-405)	
Age (years)	60 (50-67)	62 (53-68)	63 (56-71)	p=0.275
Males	52 (85.2%)	40 (72.7%)	38 (74.5%)	p=0.212
NYHA-class				
II	32 (52.5%)	36 (65.5%)	29 (56.9%)	p=0.687
III	27 (44.3%)	18 (32.7%)	20 (39.2%)	
IV	2 (3.3%)	1 (1.8%)	2 (3.9%)	
LVEF				
Mild	18 (29.5%)	18 (32.7%)	26 (51%)	p=0.091
Moderate	19 (31.1%)	21 (38.2%)	11 (21.6%)	
Severe	24 (39.3%)	16 (29.1%)	14 (27.5%)	
Comorbidities				
Hypertension	41 (67.2%)	38 (69.1%)	37 (72.5%)	p=0.828
Atrial fibrillation/flutter	26 (42.6%)	28 (50.8%)	21 (41.2%)	p=0.544
Previous MCI	3 (4.9%)	3 (5.5%)	0 (0%)	p=0.252
PAD	3 (4.9%)	5 (9.1%)	4 (7.8%)	p=0.669
Previous stroke or TIA	4 (6.6%)	5 (9.1%)	5 (9.8%)	p=0.805
Hyperlipidaemia	26 (42.6%)	25 (45.5%)	27 (52.9%)	p=0.538
Diabetes mellitus	19 (31.1%)	18 (32.7%)	13 (25.5%)	p=0.695
Previous or active smoker	49 (80.3%)	40 (72.7%)	36 (70.6%)	p=0.450
Laboratory parameters				
Creatinine (mg/dl)	1.14 (0.94-1.58)	1.11 (0.94-1.38)	1.08 (0.91-1.35)	p=0.489
eGFR, MDRD (ml/min/1.73 m^2)	66.52 (47.40-85.17)	63.77 (51.39-80.12)	70.66 (50.53-85.26)	p=0.778
eGFR, CKD-EPI (ml/min/1.73 m^2)	83.02 (73.28-94.21)	83.08 (71.69-92.05)	84.07 (74.73-91.44)	p=0.998
NT-proBNP (pg/ml)	1263.00 (473.45-3030.00)	1212.00 (403.30-2739.00)	723.30 (338.40-1331.00)	p=0.058
Phosphate (mmol/l)	1.21 (1.11-1.34)	1.06 (0.91-1.19)	0.94 (0.81-1.10)	**p<0.001**
Total calcium (mmol/l)	2.43 (2.34-2.55)	2.44 (2.37-2.49)	2.47 (2.39-2.52)	p=0.478
Albumin (g/l)	43.65 (40.33-45.95)	43.90 (41.90-45.20)	44.60 (42.40-46.80)	p=0.127
iFGF-23 (pg/ml)	68.20 (50.35-97.05)	62.30 (43.50-86.40)	63.60 (42.40-78.90)	p=0.121
cFGF-23 (RU/ml)	51.00 (21.45-156.05)	40.50 (18.00-97.50)	31.90 (16.00-82.80)	p=0.279
Magnesium (mmol/l)	0.83 (0.77-0.91)	0.83 (0.76-0.86)	0.84 (0.80-0.87)	p=0.427
CRP (mg/dl)	0.51 (0.22-0.98)	0.36 (0.15-0.72)	0.27 (0.11-0.67)	p=0.106

cFGF-23: C-terminal fibroblast growth factor 23; iFGF-23: intact fibroblast growth factor 23; HFrEF: heart failure with reduced ejection fraction; CRP: C-reactive protein; eGFR, CKD-EPI: estimated glomerular filtration rate using the Chronic Kidney Disease Epidemiology Collaboration-formula; eGFR, MDRD: estimated glomerular filtration rate using the Modification of Diet in Renal Disease-formula; MCI: myocardial infarction; NT-proBNP: N-terminal pro B-type natriuretic peptide; NYHA: New York Heart Association functional classification; LVEF: reduced left ventricular ejection fraction (mild:40-50% ejection fraction; moderate: 30-40% ejection fraction; severe: <30% ejection fraction); PAD: peripheral artery disease; T_{50}: serum calcification propensity expressed by the half-maximum transition time from primary to secondary calciprotein particles as measured by nephelometry; TIA: transient ischemic attack

Supplemental Table 3: Univariate and multivariate Cox regression analysis of phosphate, iFGF-23 and cFGF-23 for all-cause and cardiovascular mortality

	**All-Cause Mortality**				**Cardiovascular Mortality**			
	**Univariate analysis**		**Multivariate anlaysis**		**Univariate analysis**		**Multivariate anlaysis**	
	**HR (95% CI)**	**P**	**HR (95% CI)**	**P**	**HR (95% CI)**	**P**	**HR (95% CI)**	**P**
**Overall Cohort**								
Phosphate*	1.14 (0.90 – 1.46)	0.277			1.15 (0.86 – 1.53)	0.353		
Model 1			1.19 (0.94 – 1.52)	0.157			1.20 (0.90 – 1.60)	0.224
Model 2			1.12 (0.86 – 1.45)	0.398			1.08 (0.79 – 1.47)	0.647
Model 3			1.08 (0.82 – 1.43)	0.581			1.04 (0.74 – 1.45)	0.822
iFGF-23**	1.57 (1.20 – 2.05)	**0.001**			1.61 (1.17 – 2.22)	**0.004**		
Model 1			1.45 (1.09 – 1.94)	**0.012**			1.49 (1-05 – 2.10)	**0.024**
Model 2			1.29 (0.93 – 1.77)	0.122			1.24 (0.84 – 1.83)	0.27
Model 3			1.06 (0.68 – 1.64)	0.792			1.15 (0.67 – 1.95)	0.615
cFGF-23**	1.25 (1.13 – 1.38)	**<0.001**			1.29 (1.15 – 1.45)	**<0.001**		
Model 1			1.20 (1.08 – 1.34)	**0.001**			1.24 (1.10 – 1.40)	**<0.001**
Model 2			1.13 (0.99 – 1.29)	0.063			1.15 (0.99 – 1.34)	0.074
Model 3			0.97 (0.81 – 1.16)	0.735			1.03 (0.84 – 1.26)	0.794
**Ischemic HFrEF**								
Phosphate*	1.19 (0.85 – 1.66)	0.321			1.23 (0.83 – 1.81)	0.299		
Model 1			1.24 (0.89 – 1.72)	0.202			1.28 (0.88 – 1.87)	0.193
Model 2			1.27 (0.88 – 1.83)	0.197			1.30 (0.87 – 1.96)	0.204
Model 3			1.41 (0.95 – 2.11)	0.09			1.37 (0.88 – 2.15)	0.163
iFGF-23**	1.55 (1.05 – 2.29)	**0.027**			1.68 (1.08 – 2.62)	**0.020**		
Model 1			1.50 (1.00 – 2.26)	0.052			1.64 (1.03 – 2.61)	**0.036**
Model 2			1.48 (0.93 – 2.35)	0.097			1.61 (0.96 – 2.72)	0.072
Model 3			1.42 (0.72 – 2.80)	0.315			1.56 (0.73 – 3.33)	0.253
cFGF-23**	1.19 (1.03 – 1.37)	**0.018**			1.24 (1.07 – 1.44)	**0.005**		
Model 1			1.15 (0.99 – 1.34)	0.075			1.20 (1.03 – 1.41)	**0.021**
Model 2			1.08 (0.91 -1.29)	0.363			1.18 (0.97 – 1.42)	0.095
Model 3			0.85 (0.66 – 1.09)	0.207			1.04 (0.79 – 1.38)	0.772
**Non-ischemic HFrEF**								
Phosphate*	1.11 (0.78 – 1.57)	0.563			1.07 (0.69 – 1.65)	0.766		
Model 1			1.16 (0.81 – 1.65)	0.421			1.11 (0.72 – 1.73)	0.634
Model 2			0.99 (0.69 – 1.44)	0.972			0.84 (0.52 -1.34)	0.466
Model 3			0.94 (0.62 – 1.42)	0.769			0.78 (0.45 – 1.34)	0.364
iFGF-23**	1.57 (1.08 – 2.30)	**0.019**			1.52 (0.94 – 2.45)	0.087		
Model 1			1.48 (0.97 – 2.26)	0.07			1.43 (0.84 – 2.43)	0.19
Model 2			1.16 (0.72 – 1.87)	0.546			0.90 (0.49 – 1.65)	0.738
Model 3			0.83 (0.43 – 1.59)	0.573			0.76 (0.33 – 1.73)	0.509
cFGF-23**	1.34 (1.13 – 1.57)	**0.001**			1.37 (1.12 – 1.67)	**0.002**		
Model 1			1.31 (1.10 – 1.56)	**0.002**			1.35 (1.09 – 1.68)	**0.006**
Model 2			1.21 (0.97 – 1.51)	0.096			1.09 (0.84 – 1.43)	0.513
Model 3			1.00 (0.76 – 1.32)	1			0.91 (0.66 – 1.25)	0.572

The multivariate model was adjusted for the following covariates:

Model 1: age and sex;

Model 2: age, sex, smoking, systolic blood pressure, diabetes, total cholesterol, low density lipoprotein, body mass index and history of cardiovascular events;

Model 3: age, sex, race, smoking, systolic blood pressure, diabetes, total cholesterol, low density lipoprotein, body mass index, history of cardiovascular events, high sensitivity C-reactive protein, NT-proBNP, NYHA classification and eGFR (CKD-EPI)

** Hazard ratios reflect the impact of a one standard deviation increase in serum phosphate.*

*** Hazard ratios reflect the impact of doubling of FGF-23 on the original variable's scale.*

cFGF-23: C-terminal fibroblast growth factor 23; iFGF-23: intact fibroblast growth factor 23; NT-proBNP: N-terminal pro B-type natriuretic peptide; NYHA: New York Heart Association functional classification; eGFR (CKD-EPI): estimated glomerular filtration rate using the Chronic Kidney Disease Epidemiology Collaboration-formula; HFrEF: heart failure with reduced ejection fraction; HR: hazard ratio

Supplemental Figure 1: Kaplan-Meier estimates for overall survival according to iFGF-23 tertiles for the whole cohort (A, p=0.001), patients with ischemic heart failure with reduced ejection fraction (HFrEF; B, p=0.148) and patients with non-ischemic HFrEF (C, p=0.005)

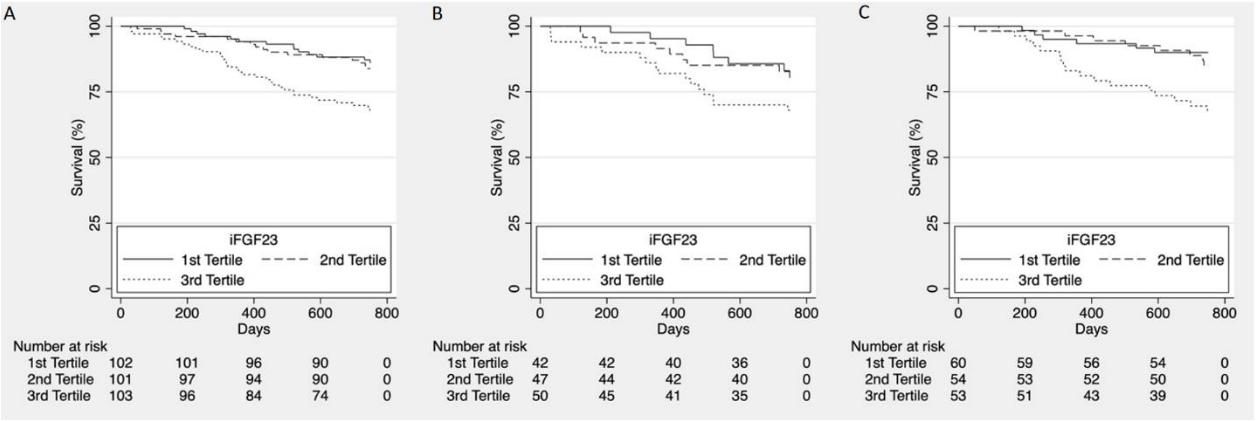

iFGF-23: intact fibroblast growth factor 23

Supplemental Figure 2: Kaplan-Meier estimates for overall survival according to cFGF-23 tertiles for the whole cohort (A, $p \leq 0.001$), patients with ischemic heart failure with reduced ejection fraction (HFrEF; B, p=0.037) and patients with non-ischemic HFrEF (C, p=0.001)

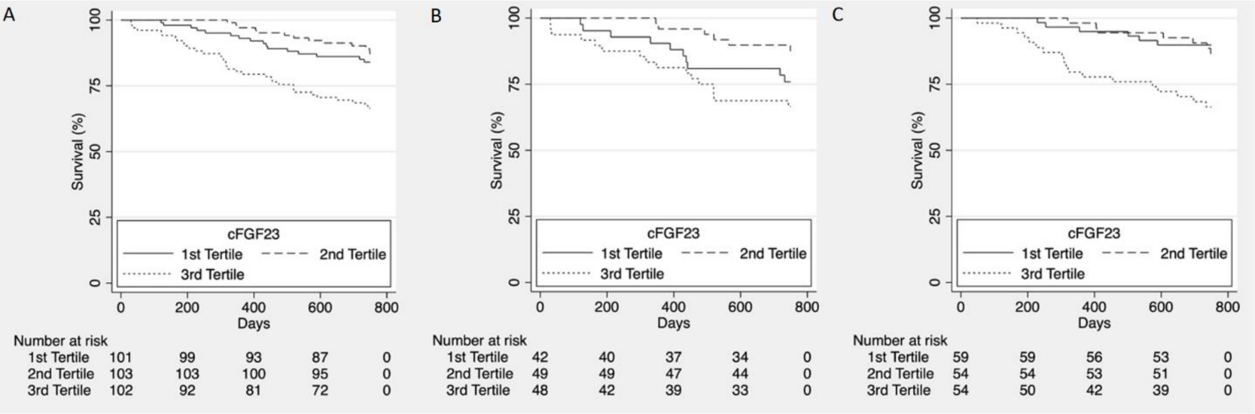

cFGF-23: C-terminal fibroblast growth factor 23

Supplemental Figure 3: Kaplan-Meier estimates for cardiovascular survival according to iFGF-23 tertiles for the whole cohort (A, p=0.005), patients with ischemic heart failure with reduced ejection fraction (HFrEF; B, p=0.107) and patients with non-ischemic HFrEF (C, p=0.040)

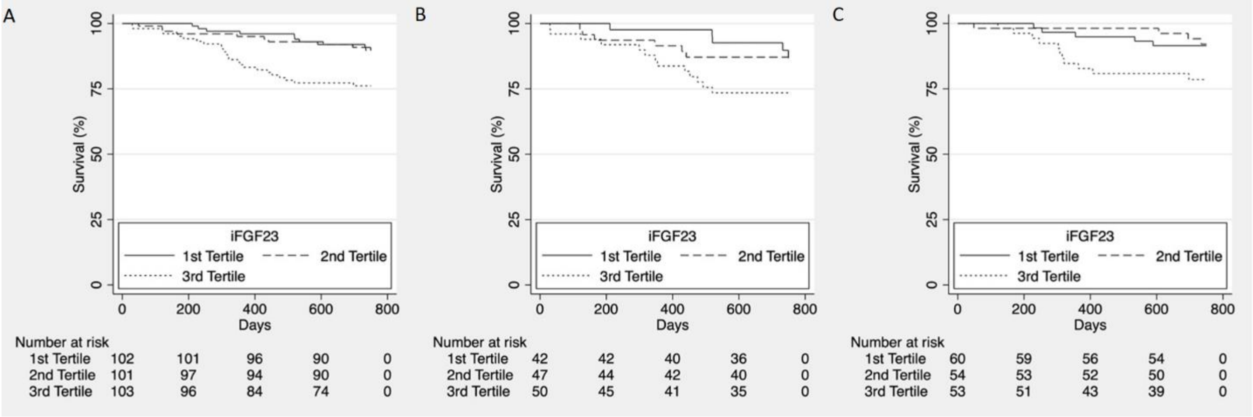

iFGF-23: intact fibroblast growth factor 23

Supplemental Figure 4: Kaplan-Meier estimates for cardiovascular survival according to cFGF-23 tertiles for the whole cohort (A, p≤0.001), patients with ischemic heart failure with reduced ejection fraction (HFrEF; B, p=0.054) and patients with non-ischemic HFrEF (C, p=0.009)

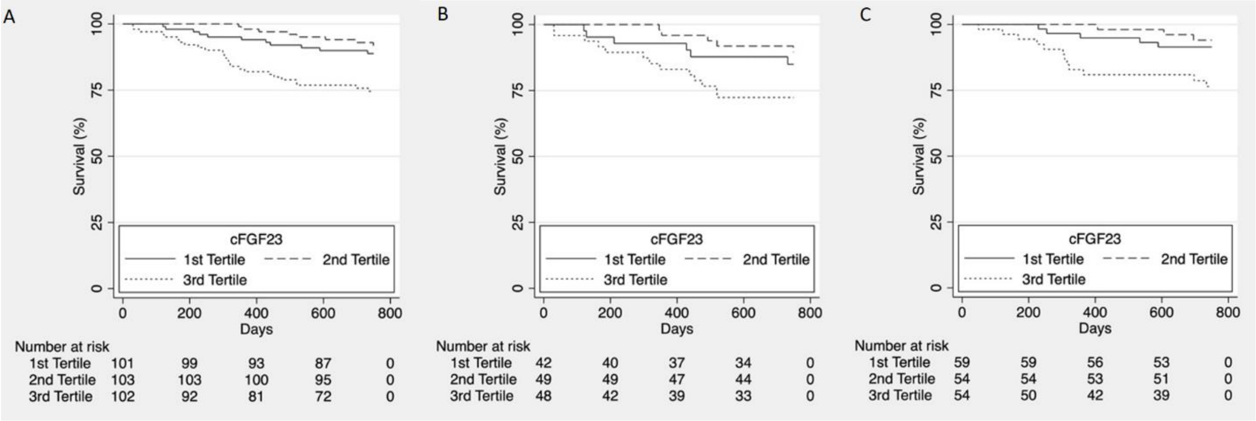

cFGF-23: C-terminal fibroblast growth factor 23
